# A 33-Year-Old Plant Sample Contributes the First Complete Genomic Sequence of *Potato Virus U*

**DOI:** 10.1128/MRA.01392-18

**Published:** 2018-12-13

**Authors:** Ian P. Adams, Neil Boonham, Roger A. C. Jones

**Affiliations:** a Fera Science Ltd., Sand Hutton, York, United Kingdom; b School of Natural and Environmental Sciences, Newcastle University, Newcastle upon Tyne, United Kingdom; c Institute of Agriculture, Faculty of Science, University of Western Australia, Crawley, Western Australia, Australia; d Department of Primary Industries and Regional Development, South Perth, Western Australia, Australia; Indiana University, Bloomington

## Abstract

A *Potato virus U* isolate detected in a Peruvian potato sample collected in 1977 produced the first genome sequence of this virus. When this genome sequence was compared with those of other nepoviruses, the amino acid sequences of RNA1 and RNA2 were most similar to those of subgroup C nepoviruses.

## ANNOUNCEMENT

From a sample collected during a 1977 survey of subsistence crops at an altitude of 3,600 m in the Comas Valley, Department of Junin in the Peruvian Andes, a virus was isolated from plants of potato (Solanum tuberosum subsp. *andigena*) showing bright yellow leaf markings. The virus was mechanically transmissible to 44 species from 7 plant families. However, it proved difficult to reestablish systemic infection with it in potato plants, so it may predominantly infect potato roots and rarely invade the infected plants systemically or, alternatively, be mostly associated with other as yet unknown plant species, only infecting potato occasionally. The virus was serologically unrelated to 17 other nepoviruses, including *Arracacha virus A* (1) and *Potato black ringspot virus* (2) from Peru (3, 4). Following characterization using electron microscopy, density centrifugation, and serology, it was identified as a member of the nepovirus genus and named *Potato virus U* (PVU) (3, 4). In 1978, PVU-infected leaf samples were dried over silica gel in Peru, sealed in glass vials (code name UC), and sent to the United Kingdom. The virus was recovered there and studied further before being freeze-dried in glass vials (isolate code UC) in 1984. These vials currently form part of the plant virus collection at Fera Science Ltd. (York, United Kingdom).

In this study, the methods used resemble those described previously (5–7). In 2017, using an RNAeasy kit (Qiagen, UK), a total RNA extract was obtained from the freeze-dried, PVU-infected leaves. A ScriptSeq complete plant leaf kit (Illumina, USA) was utilized to obtain an indexed plant ribosome-subtracted sequencing library from the total RNA following the manufacturer’s specifications. A 600-cycle v3 kit was then employed to sequence this library on a MiSeq instrument (Illumina) with other libraries. The paired-end reads were 3′ trimmed to a quality score of 20 with Sickle in paired-end mode (8). These reads were assembled with Trinity v2 with the maximum memory allocation set to 99 gigabytes of RAM, and the process allocated 64 central processing units (9). The resulting contigs were compared to the GenBank nonredundant (nr) and nucleotide databases with BLAST+ (v2.2.29) (10). Reads of viral origin were extracted with the extract reads function in MEGAN (Community Edition 6.10.2) (11). Two contigs, 5,963 and 4,799 nucleotides (nt) long, were assembled and shown by comparison to other nepovirus genomes to represent the RNA1 and RNA2 components of the PVU genome.

The PVU RNA1 encoded a putative 218-kDa polyprotein with an amino acid identity resembling those of the subgroup C nepoviruses *Soybean latent spherical virus* (67%) (12) and *Blueberry latent virus* (54%) (13). The PVU RNA2 encoded a putative 170-kDa polyprotein with 44% and 60% amino acid identity to *Blueberry latent virus* (13) and *Soybean latent spherical virus* (12), respectively. A search of the Conserved Domains Database (CDD) (14) with the predicted polyprotein revealed the presence of *Nepovirus* coat protein domains. These findings, the earlier serology and electron microscopy studies (3, 4), and the absence of any significant homology between the 3′ untranscribed regions (UTRs) of its RNA1 and RNA2 molecules show that PVU meets the membership criteria of subgroup C within the genus *Nepovirus* (15).

### Data availability.

The sequences described here were deposited in GenBank under accession numbers MH716806 (RNA1) and MH716805 (RNA2). Raw data were deposited in the SRA under accession number SAMN10081142, part of BioProject number PRJNA491634.
